# Genome-wide association study between copy number variation and feeding behavior, feed efficiency, and growth traits in Nellore cattle

**DOI:** 10.1186/s12864-024-09976-8

**Published:** 2024-01-11

**Authors:** Lorena F. Benfica, Luiz F. Brito, Ricardo D. do Bem, Henrique A. Mulim, Joseph Glessner, Larissa G. Braga, Leonardo S. Gloria, Joslaine N. S. G. Cyrillo, Sarah F. M. Bonilha, Maria E. Z. Mercadante

**Affiliations:** 1 Department of Animal Sciences, Purdue University, 270 S. Russell Street, West Lafayette, IN 47907 USA; 2 Department of Animal Science, Faculty of Agricultural and Veterinary Sciences, Sao Paulo State University, Jaboticabal, SP Brazil; 3 Perelman School of Medicine, University of Pennsylvania, Philadelphia, PA USA; 4 Center for Applied Genomics, Children’s Hospital of Philadelphia, Philadelphia, PA USA; 5 Institute of Animal Science, Sertaozinho, SP Brazil

**Keywords:** *Bos indicus*, Bovine genome, SNP panel, Structural variants

## Abstract

**Background:**

Feeding costs represent the largest expenditures in beef production. Therefore, the animal efficiency in converting feed in high-quality protein for human consumption plays a major role in the environmental impact of the beef industry and in the beef producers’ profitability. In this context, breeding animals for improved feed efficiency through genomic selection has been considered as a strategic practice in modern breeding programs around the world. Copy number variation (CNV) is a less-studied source of genetic variation that can contribute to phenotypic variability in complex traits. In this context, this study aimed to: (1) identify CNV and CNV regions (CNVRs) in the genome of Nellore cattle (*Bos taurus indicus*); (2) assess potential associations between the identified CNVR and weaning weight (W210), body weight measured at the time of selection (WSel), average daily gain (ADG), dry matter intake (DMI), residual feed intake (RFI), time spent at the feed bunk (TF), and frequency of visits to the feed bunk (FF); and, (3) perform functional enrichment analyses of the significant CNVR identified for each of the traits evaluated.

**Results:**

A total of 3,161 CNVs and 561 CNVRs ranging from 4,973 bp to 3,215,394 bp were identified. The CNVRs covered up to 99,221,894 bp (3.99%) of the Nellore autosomal genome. Seventeen CNVR were significantly associated with dry matter intake and feeding frequency (number of daily visits to the feed bunk). The functional annotation of the associated CNVRs revealed important candidate genes related to metabolism that may be associated with the phenotypic expression of the evaluated traits. Furthermore, Gene Ontology (GO) analyses revealed 19 enrichment processes associated with FF.

**Conclusions:**

A total of 3,161 CNVs and 561 CNVRs were identified and characterized in a Nellore cattle population. Various CNVRs were significantly associated with DMI and FF, indicating that CNVs play an important role in key biological pathways and in the phenotypic expression of feeding behavior and growth traits in Nellore cattle.

**Supplementary Information:**

The online version contains supplementary material available at 10.1186/s12864-024-09976-8.

## Background

Brazil is one of the largest beef exporters in the world, with a cattle population composed of about 80% of Nellore (*Bos taurus indicus*) or Nellore composite breed animals [1]. With a rapid increase in the world population and reduction in poverty, beef consumption is expected to increase from 60 to 130 million tons by 2050, and ~ 70% of this growth is projected to be provided by beef production systems from tropical and subtropical regions, including Brazil [2]. To meet the world’s growing beef demand and reduce the environmental impact of the industry, especially in developing countries, there is an urgent need to develop more efficient breeding strategies for genetically improving tropically-adapted cattle raised in pasture-based production systems. Since feed represents the largest costs in beef production and is a major determinant of beef cattle producers’ profitability [3], improving cattle feed efficiency has been considered as a strategic and major breeding goal in worldwide beef cattle breeding programs [4–7]. Additionally, feeding behavior traits are associated with feed efficiency and growth traits [8, 9], and could be used as auxiliary traits for further improving beef cattle feed efficiency.

The sequencing of the cattle genome has led to the discovery of thousands of single nucleotide polymorphism (SNP) markers [10], which are common variants of individual nucleotide sequences that are frequently observed in the population (> 1%). Following the sequence of the cattle genome, various SNP panels containing thousands of markers with great genome coverage were developed [10]. In addition to providing information on individual SNPs, SNP panel data can also be used for identifying a form of genomic structural variation known as copy number variation (CNV; [11]).

The CNVs are a less-studied source of genetic variation that can influence phenotypic variability in complex traits. They can be defined as structural variations in an individual’s genome in the form of losses or gains of DNA fragments ranging from 1 kb to several mega-bases in comparison to the reference genome of the species [12–14]. Additionally, CNVs are polymorphic genetic markers that can be inherited across generations [15]. CNVs can be detected using various platforms and sequencing tools, including array comparative genome hybridization (aCGH), single nucleotide polymorphism (SNP) panels, and next-generation sequencing (NGS) tools [16–18]. One of the most used data sources has been SNP panels as they are already generated for genomic selection purposes in commercial breeding programs [19].

Compared to individual SNPs, CNVs cover wider chromosomal regions [20], which contribute to changes in genome structure, alteration in regulation or gene dosage, exposure of recessive alleles, and alterations in gene expression, and consequently, phenotypic variability in complex traits [21–23]. CNVs can also have unique functional consequences not producible by SNPs. For instance, duplications can increase gene dosage while deletions can eliminate regulatory elements [24]. In addition, the lack of linkage disequilibrium between SNPs and 25% of the detected CNVs indicate that CNVs contain information not captured solely based on SNP information [25, 26]. Therefore, CNV is an additional source of information to explain the genetic variance of complex traits not accounted for by SNPs alone [26].

Many studies investigating CNVs have been carried out over the past few years. These studies have shown that these structural variations are major contributors to genetic diversity and phenotypic variability in many species, including humans [27–31], birds [32], pigs [23, 33], sheep [34–36], and cattle [25, 37–39]. However, there is limited information on how CNVs contribute to the phenotypic variation in traits related to feed efficiency, feeding behavior, and growth in cattle, especially in Zebu cattle (*Bos taurus indicus*) such as in the Nellore breed– the major Zebu breed in Brazil. Therefore, the main objectives of this study were to: (1) identify CNV and CNV regions (CNVR) in the genome of a Nellore cattle population; (2) assess potential associations between the identified CNVR and weaning weight adjusted to 210 days (W210), body weight measured at the time of selection (WSel), average daily gain (ADG), dry matter intake (DMI), residual feed intake (RFI), time spent at the feed bunk (TF), and frequency of visits to the feed bunk (FF) traits; and, (3) perform functional genomic annotation of the associated CNV regions (CNVRs).

## Results

The descriptive statistics for the phenotype and the adjusted phenotypes used for the analyses are presented in Additional File 1.

### Copy number variation and CNVR detection

Initially, 8,170 individual CNVs were detected in 1,222 samples. After the quality control, 3,161 CNVs located in the autosomal chromosomes from 620 samples remained for further analyses, with a mean number of CNVs per animal equal to five (range from 1 to 35). Out of the CNVs identified, 1,401 were deletions and 1,760 were duplications. The length of the CNVs ranged from 4,974 bp to 2,191,266 bp with an average length of 176,335 ± 181,997 bp. No CNVs were detected on *Bos taurus* autosomes (BTA) BTA27 and BTA28. However, BTA6 exhibited the highest number of CNVs, with a maximum of 296 CNVs. On the other hand, BTA24 had the lowest number (*n* = 3) of CNVs.

**Fig. 1:**
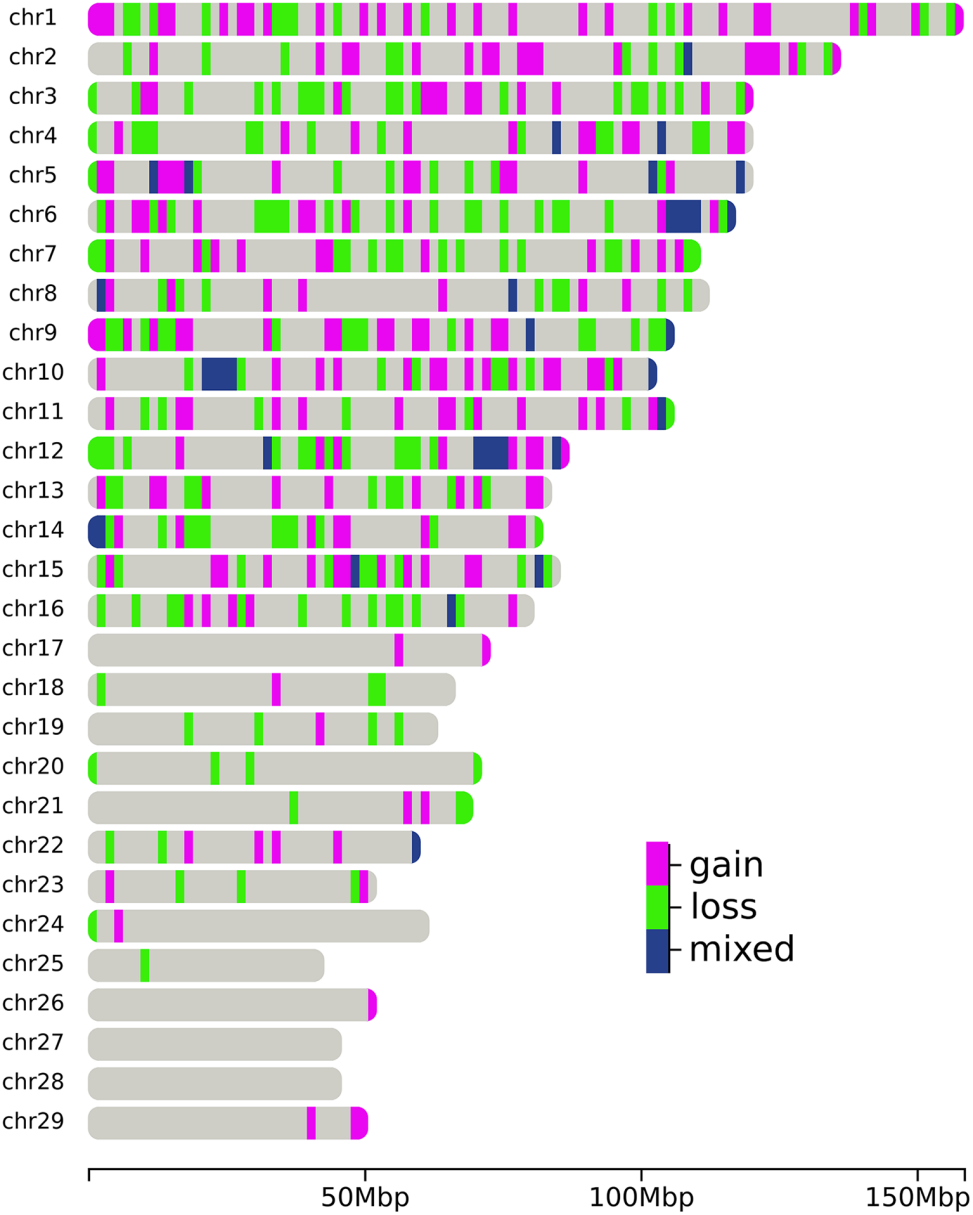
Distribution of copy number variation regions (deletions, duplications, and mixed type) by chromosome

The 3,161 CNVs remaining after quality control were used to infer CNVR by merging CNV with at least 1 bp overlap. Thus, 561 CNVRs were identified, in which the average CNVRs length was 176,866 ± 263,706 bp and they ranged from 4,973 bp to 3,215,394 bp. Among these CNVRs, 256 correspond to genome deletions, 245 to duplications, and 60 to mixed pattern (i.e., the same chromosomal segment was a deletion or duplication in different animals). The deletion-to-duplication ratio was 1.04. Thirty-nine CNVRs were identified in at least 1% of the studied population. The number and proportion of chromosomes covered by CNVRs varied considerably and no CNVRs were identified on BTA27 and BTA28 (Fig. 1; Table 1). BTA1 had the largest number of CNVRs (*n* = 49), which covered 4.08% of the chromosome, and BTA12 presented the highest coverage of a chromosome sequence (10.2%) with 41 CNVRs. The CNVRs inferred in our study covered 99,221,894 bp of the autosomal genome sequence, which corresponds to 3.99% of the cattle genome size.

**Table 1:** Chromosome distribution of all 561 copy number variation regions (CNVRs) detected in the Nellore genome

Chr^a^	Chr length (bp)	Number of CNVRs	CNVR length (bp)	%^b^
BTA1	158,534,110	49	6,469,894	4.08
BTA2	136,231,102	35	5,392,406	3.96
BTA3	121,005,158	40	5,802,111	4.79
BTA4	120,000,601	33	4,625,409	3.85
BTA5	120,089,316	30	8,863,760	7.38
BTA6	117,806,340	43	9,353,904	7.94
BTA7	110,682,743	39	4,660,233	4.21
BTA8	113,319,770	21	3,178,109	2.80
BTA9	105,454,467	39	5,500,040	5.21
BTA10	103,308,737	23	5,660,780	5.48
BTA11	106,982,474	26	4,572,944	4.27
BTA12	87,216,183	41	8,878,934	10.2
BTA13	83,472,345	22	2,443,887	2.93
BTA14	82,403,003	26	4,704,971	5.71
BTA15	85,007,780	29	4,596,564	5.41
BTA16	81,013,979	20	2,809,390	3.47
BTA17	73,167,244	3	2,360,049	3.22
BTA18	65,820,629	4	579,684	0.88
BTA19	63,449,741	6	914,327	1.44
BTA20	71,974,595	4	1,039,708	1.44
BTA21	69,862,954	6	2,422,071	3.47
BTA22	60,773,035	8	1,785,240	2.94
BTA23	52,498,615	5	899,028	1.71
BTA24	62,317,253	2	127,751	0.20
BTA25	42,350,435	2	803,807	1.89
BTA26	51,992,305	2	454,688	0.87
BTA27	45,612,108	0	0	0
BTA28	45,940,150	0	0	0
BTA29	51,098,607	3	322,205	0.63
**Total**	**2,489,385,779**	**561**	**99,221,894**	**3.99**

^a^Chromosome

^b^The percentage of the chromosome covered by the copy number variation regions

### Gene annotation, enrichment analyses, and QTL identification

Association analyses between the traits and the discovered CNVs of 620 animals led to the identification of 17 CNVRs significantly associated with at least 1 of the evaluated traits (*P* < 0.0005). The 17 regions represent 2 deletions, 9 duplications, and 6 mixed, distributed across 14 chromosomes with an average length of 273,250 ± 209,907 bp (Table 2). Out of the 17 CNVRs, 16 were associated with FF and 1 CNVR was associated with DMI and 12 were found in gap of the reference assembly ARS-UCD1.2 (Additional file 2). No CNVRs were significantly associated with the other traits.

**Table 2:** Copy number variation regions associated with growth, feed efficiency, and feeding behavior in Nellore cattle

CNVR^a^	Chr^b^	Start (bp)	End (bp)	Type	Trait^c^
CNVR1	BTA2	135,110,420	135,653,313	Mixed	FF
CNVR2	BTA5	117,080,458	117,820,070	Deletion	FF
CNVR3	BTA6	116,755,758	117,164,372	Duplication	FF
CNVR4	BTA7	10,092,268	10,174,209	Duplication	FF
CNVR5	BTA7	42,951,015	43,292,715	Mixed	FF
CNVR6	BTA7	43,359,066	43,823,809	Mixed	FF
CNVR7	BTA8	15,562,312	15,781,720	Duplication	FF
CNVR8	BTA8	38,356,510	38,610,355	Duplication	FF
CNVR9	BTA8	85,996,187	86,508,867	Deletion	DMI
CNVR10	BTA9	2,637,837	2,700,411	Mixed	FF
CNVR11	BTA9	16,366,613	16,894,948	Duplication	FF
CNVR12	BTA9	15,312,685	15,469,154	Duplication	FF
CNVR13	BTA12	73,233,249	73,770,215	Mixed	FF
CNVR14	BTA12	74,302,958	74,578,587	Mixed	FF
CNVR15	BTA12	64,618,237	64,736,496	Duplication	FF
CNVR16	BTA13	12,552,408	12,829,168	Duplication	FF
CNVR17	BTA26	51,032,219	51,267,717	Duplication	FF

^a^Copy number variation region (CNVR) significantly (*P* < 0.005) associated with the traits

^b^Chromosome

^c^DMI: dry matter intake; FF: feed frequency

Functional enrichment analyses were performed to obtain broad functional insights into the set of genes significantly associated with the CNVRs for each trait. Gene Ontology enrichment analyses revealed 19 processes for FF, which are categorized as 10 biological processes, three cellular components, four molecular functions, and two metabolic pathways (*p* < 0.05, as shown in Additional file 3). No GO enrichment processes were identified for WSel, W210, ADG, DMI, RFI, TF.

In total, 73 previously-reported quantitative trait loci (QTL) overlapped with the genomic regions associated with DMI and FF (Additional file 4). The number of overlapping QTLs were 2 for DMI and 71 for FF. The QTL identified span a wide range of trait types, including meat and carcass, milk, reproduction, and production. These QTL are also associated with many important traits, including marbling score, fat cover carcass, body height, body weight, carcass weight, dry matter intake, and average daily gain.

## Discussion

All the growth, feed efficiency, and feeding behavior traits included in this study are heritable with heritability estimates ranging from 0.17 ± 0.03 (RFI) to 0.51 ± 0.06 (TF) [9]. Considering that CNVs are genomic alterations that can affect gene expression and, consequently, influence an individual’s phenotype [22, 23] and that heritability indicates the proportion of phenotypic variation in a population attributable to genetic factors, a portion of the heritability of a given trait may be explained by genomic structural variations, such as CNVs. For instance, high heritability for specific traits implies that a substantial portion of the trait’s phenotypic variability is attributable to genetic factors, which may include CNVs. This highlights the importance of considering these genomic alterations in the context of inheritance and phenotypic variability within populations.

### Identification of CNV and CNVR

After applying quality control measures, approximately 60% of the initially detected CNVs were excluded from further analyses and many factors might be associated with this substantial reduction in the number of CNVs. For instance, the use of a rigorous quality control aiming to minimize false-positive CNV calls could result in the exclusion of true CNVs. Additionally, the design of the SNP panel itself could have played a key role in the reduction in the number of CNVs identified. The distribution of markers across the genome may not have fully captured certain CNVs, particularly those located in regions less represented by the SNP panel. Furthermore, the gap between markers could have also influenced the detection, as it might ignore smaller CNVs located within these gaps, or even the exclusion of larger CNVs when the gap between markers is too large.

After the quality control, 3,161 CNVs (1,401 deletions and 1,760 duplications) with an average length of 176,335 ± 181,997 bp remained for further analyses. Despite the number of CNVs corroborating with values reported in the literature, there are often significant variability in the results of CNV analyses from different studies even within the same species. For instance, Butty et al. [39], while studying a population of 10,682 Holstein animals using SNP panels of different densities (Bovine HD Beadchip HD, Genome Profiler Bovine 150 K, Genome Profiler Bovine HD, BovineSNP50, and Genome Profiler Bovine 50 K), identified an average of four CNVs per animal and a total of 23,256 CNVs with an average length of 168,520 bp. Peripolli et al. [40], studying whole-genome re-sequencing from 36 animals of different breeds, reported 7,285 CNVs in the population, with an average of 607.08 CNVs per animal, and an average length of 28,300 bp. Lemos et al. [38], studying 3,794 Nellore breed animals genotyped with a high density (HD) SNP panel and without adopting any quality control, identified 399,361 CNVs with an average length of 54,744 bp. Hou et al. [41], working with a population of 427 Angus reported 2,724 CNVs with an average of six CNVs per animal. These differences observed can be explained by differences in the data and methodologies used, including (1) the use of different platforms and methods (based on Comparative Genomic Hybridization, SNP-array, and Next Generation Sequencing); (2) the software utilized for the analyses; (3) quality control metrics and thresholds; (4) density of the genotyping SNP panels; and, (5) sample size [21, 42–44]. Despite the fact that all four studies mentioned above, and the current study focused on cattle, they all utilized different genotyping platforms, represented different populations, and differed on the sample sizes. These differences could justify the results obtained and make direct comparisons among studies unfair. It is important to acknowledge the impact of these differences when interpreting the findings. However, despite these discrepancies among studies, we observed some trends indicating that higher density panels tend to be associated with a greater number of CNVs and a shorter CNVs on average.

Deletion and duplication are genetic events that involve the number of copies of a particular DNA sequence. Deletion refers to the loss of a DNA segment from a chromosome and can impact phenotypic expression by interrupting genes and causing loss of biological functions [45]. Duplication refers to the process in which a segment of DNA is duplicated, resulting in additional copy of that sequence. Duplications are usually reported associated with digestion processes, lactation, reproduction, and immune system such as antigen processing and major histocompatibility genes in the cattle genome [46]. More duplications than deletions were identified in the present study, which is in agreement with Ladeira et al. [35] who reported 322 deletions and 835 duplications in sheep, and Liu et al. [47] who observed more duplication type (*n* = 11) CNVs as compared to deletion (*n* = 9) CNVs in Angus cattle. These findings deviate from the prevailing pattern observed in the literature regarding CNV surveys in animals, where deletions tend to be more frequent than duplications [23, 39, 40, 48].

The average length of the CNVs identified in the present study is in line with the distance between markers on the GGP 75 K panel (107,700 bp; at least three SNPs were required to be considered as a CNV). The distribution of CNVs was not uniform across the cattle autosomes. This observation may be related with the different mechanisms of CNV formation, such as nonallelic homologous recombination (NAHR), fork stalling and template switching (Fostes, a mechanism based on DNA replication error), and nonhomologous end-joining (NHEJ), once each mechanism would take place more often in certain genomic regions than others [20].

The proportion of the genome covered by CNVRs (3.99%) was consistent with values reported in the literature, which range from 0.68 to 9.43% [20, 37, 39, 47]. The variation in genome coverage observed across studies can be attributed to the specific SNP panels used, which, similarly to CNV detection, can potentially affect the sensitivity of CNV detection. When a low or medium-density SNP panel is used, there is a lower number of potential breakpoints compared to higher density SNP panels. As a result, the CNVs identified tend to be longer and cover a larger portion of the genome [39].

### Association between CNVR and growth, feed efficiency, and feeding behavior traits

Copy number variation has the potential to modify gene expression, as deletions or duplications of gene segments, either partial or complete, can disrupt gene function and lead to phenotypic changes [49]. Consequently, identifying CNVRs that overlap with genes becomes a crucial step in assessing their functional impact. In this study, we further investigated the CNVRs identified based on the ARS-UCD1.2 cattle genome assembly. Remarkably, many CNVRs were associated with growth and feeding behavior, indicating that these CNVRs may play a role in influencing the phenotypic expression of these traits.

In this study, approximately 71% of the CNVRs significantly associated with the studied traits overlapped with Ensembl genes. This observation indicates that CNVRs often occur in gene-rich regions and suggests that these CNVRs could potentially have functional implications. This is because they might influence the expression or regulation of nearby genes and possibly contribute to the phenotypic variations associated with important traits. In total, 95 genes overlapped with these genomic regions and 83 of them were classified as protein-coding genes. A similar trend was observed in previous studies in pigs [50] and beef cattle [46], where CNVRs were also found to be concentrated in protein-coding genes. Protein-coding genes are segments of DNA that serve as template for transcription of the DNA into RNA sequences and the complementary chain [51, 52]. The importance of this type of genes lies in their ability to direct the synthesis of specific proteins. They are also essential to the task of translating the information in the sequence of the genome into biologically relevant knowledge and can affect dosage-sensitive genes [51]. Therefore, the presence of CNVRs in protein-coding genes may be relevant to explain the associations with the studied traits.

The lack of significantly identified CNVRs associated with WSel, W210, ADG, RFI, and TF traits may be linked to the limited number of genotyped animals with these phenotypes. Increasing the sample size for future studies could enhance the precision in identifying genetic variants associated with the phenotypes of interest and increase the accuracy and reliability of genetic variations associated with the traits under investigation. FF was the trait with the highest number of significant CNVRs and overlapped with important genes and QTLs. FF was also associated with some QTL related to dry matter intake, body weight and bovine respiratory disease (BRD) susceptibility in cattle. These are important traits, since BRD is one of the most common and costly disease of feedlot cattle, and has a negative impact on ADG, where calves diagnosed with BRD tend to have lower ADG compared with healthy animals [53, 54]. Therefore, the genetic associations between FF with BRD susceptibility, dry matter intake, and body weight QTLs highlight the interaction between genetic factors, feeding behavior, and overall cattle performance, with significant implications for the cattle industry. Additionally, significant biological processes associated with FF were identified, particularly related to the activation of GTPase activity (GO:0090630), GMP catabolic process (GO:0046038), and cellular response to mechanical stimulus (GO:0071260).

The CNVR12 was significantly associated with FF and overlapped with *MYO9A* gene. This is a gene member of the myosin superfamily that is related to ATPase activity. ATPase activity is essential for many cellular processes, plays a crucial role in cellular energy metabolism, and is involved in a wide range of physiological processes. The CNVR17, located in the chromosome 26 overlapped with five genes, including *INPP5A* and *MRPL13*. *INPP5A* is gene that code a protein responsible for mobilizing intracellular calcium and acts as a second messenger mediating cell responses to several stimulations [55]. The *MRPL13* gene is a component of the mitochondrial ribosomal protein (MRP) family. The MRP are synthesized in the cytoplasm before being transported into the mitochondria for the purpose of mitochondrial ribosome assembly. MRP is vital for mitochondrial oxidative phosphorylation and plays a significant role in the regulation of apoptosis-inducing factors, and an alteration in the MRP expression could result in a range of disorders, including mitochondrial metabolic disorders and cellular dysfunction [56]. Although no studies have reported a direct relationship between the *MRPL13* gene and feeding behavior, it is important to highlight that the mitochondria are cellular organelles responsible for energy conversion and adenosine triphosphate (ATP) production in eukaryotic cells [57]. In addition to their function in energy metabolism, they play an important role in diverse cellular processes, such as apoptosis [58] and aging [59]. Therefore, it is plausible that the *MRPL13* gene, being involved in mitochondrial function and cellular metabolism, may have indirect implications for the organism’s response to feeding behavior, and consequently, feed efficiency and growth.

Feeding behavior is a complex process that encompasses a multitude of psychological factors, neuronal mechanisms, and metabolic processes. Thus, while none of the identified genes have been directly associated with feeding behavior traits yet, the fact that many of them are linked to metabolic activities might suggest a relationship between these genes and the FF. These novel findings highlight the importance of developing a reference genome for Nellore cattle and performing detailed functional annotation of the Nellore cattle genome.

Despite the findings obtained, there are also inherent limitations in this study. These limitations provide a solid foundation for identifying research areas that require further investigation and pave the way for more comprehensive and in-depth studies in the future. One limitation of this study is that the current study lacks detailed information regarding the storage and processing procedures of all utilized DNA samples. This stands as a crucial aspect as it can significantly impact the detection of CNVs. Variations in the DNA source have the potential to influence the genetic material’s integrity, while the processing methods such as DNA extraction, storage, or amplification might affect the quality of the genomic data acquired [60], potentially impacting the accuracy of genetic variations identification. Furthermore, low quantity and/or quality DNA samples can lead to a higher number of genotyping errors [61].

Another important point is the relatively small number of animals, which, although larger than many previous studies, may still have impacted the results reported here. Additionally, the use of the reference genome ARS-UCD1.2 could also be a limitation that affected the results, as it is based on the genome of a *Bos taurus taurus* (Taurine) animal of the Hereford breed, while the animals in the present study are *Bos taurus indicus* (Zebu) of the Nellore breed. Therefore, future studies could take these potential factors into consideration for a more comprehensive understanding of the CNVs in Zebu cattle. Furthermore, validation of CNVs and CNVRs were not performed in this study. Validation studies are important to ensure the accuracy and reliability of the CNVs detected. One alternative is to use Whole Genome Sequencing (WGS) data that can provide a more comprehensive overview of the entire genome and tends to be more sensitivity for the detection of structural and complex variants in the genome [62]. Therefore, WGS could be a powerful tool for CNV validation in future studies.

The results of the present study have significant implications and can have several practical applications. Based on the findings, one possible application is the creation of SNP panels with a higher number of markers in the regions with large incidence of CNVRs. This would allow more comprehensive and refined genomic analyses, providing a more detailed understanding of the genetic variations in those regions. Additionally, an alternative approach to consider in genomic prediction is the differential weighting of SNPs located within CNVRs. A potential method for this is the use of the Weighted Single-step Genomic BLUP procedure, as proposed by Wang et al. [63], that is an iterative process involving updates of SNP solutions with appropriate weights. Incorporating differential weighting of SNPs within genomic models could enable a more realistic representation of the actual distribution of SNP effects, with a particular emphasis on CNVRs with larger effects, and could potentially improve the accuracy of genomic prediction of breeding values [26].

## Conclusion

This study aimed to investigate CNVs and CNVRs in the genome of a Nellore cattle population and explore their associations with growth, feed efficiency, and feeding behavior traits. A total of 3,161 CNVs and 561 CNVRs were identified and characterized within the Nellore cattle genome. The results revealed that some CNVRs are significantly associated with the traits analyzed, showing the potential influence of structural genome variations on economically relevant traits in Nellore cattle. The functional annotation of the associated CNVR revealed some important genes that may be related to the expression of the traits studied. Various QTLs overlapping with the CNVRs identified are related with growth, feed efficiency, and feeding behavior traits in Nellore cattle.

## Materials and methods

No Animal Care Committee approval was necessary for the purposes of this study, as all information required was obtained from pre-existing databases.

### Animals and phenotypic datasets

Performance records were obtained from 1,338 animals born from 1983 to 2020, which belong to the Nellore cattle herd of the Institute of Animal Science (IZ), Sertãozinho, SP, Brazil. The analyzed traits were weaning weight adjusted for 210 days of age (W210), body weight measured at the time of selection (WSel), average daily gain (ADG), dry matter intake (DMI), residual feed intake (RFI), time spent at the feed bunk (TF), and frequency of visits to the feed bunk (FF).

Weaning weight adjusted for 210 days of age was calculated based on the weight gain between birth and weaning, using the following equation:

$${\rm\text{W}}210 = \left( {{\text{WW - BW} \over \text{AAW}}} \right){\rm{*}}210 + {\rm\text{BW}}$$

where W210 is the weaning weight adjusted for 210 days of age; WW is the weaning weight; BW is the birth weight; and AAW is the age of the animal at weaning. WSel is the postweaning weight adjusted to 378 days of age for males in feedlot performance tests and postweaning weight adjusted to 550 days of age for females on pasture.

Dry matter intake (DMI) and average daily gain (ADG) were obtained for males and females, which participated in 21 performance tests after weaning with a minimum of 21 days of adaptation to the diet and facilities and 86 ± 13 test days. The animals started the test at the age of 293 ± 42 days and remained in individual or collective pens equipped with the Vytelle LLC® (Vytelle LLC, Calgary, AB, Canada) or Intergado® (Contagem, Minas Gerais, Brazil) electronic monitoring system. The animals had *ad libitum* access to diet and water. The diet was formulated for an ADG of 1.1 kg. Body weights were recorded at a maximum interval of 28 days.

The DMI was obtained as the average of all valid days of intake multiplied by the dry matter content of the diet offered each week. ADG was calculated as the linear regression coefficient of weights as a function of days on test:

$$\text{y}_\text{i}= \alpha + \beta \times \text{DOT}_\text{i}+ \text{e}_\text{i},$$

where **y**_**i**_ is the animal’s weight in the i^th^ observation; **α** is the intercept corresponding to the initial weight; **β** is the linear regression coefficient corresponding to ADG; **DOT** is days of test; and **e**_**i**_ is the random error. RFI was estimated as the residual of the linear regression equation of DMI on ADG and BW^0.75^ (Koch et al., 1963). Feeding behavior data was only available for males kept in collective pens equipped with Vytelle LLC® (Vytelle LLC, Calgary, AB, Canada) in 12 feeding tests. The electronic trough systems were configured to scan the electronic identification tags of animals entering the trough every 1.0 to 6.3 s. The start of a meal event is defined when the tag of an animal was identified by the system. The meal event ends when the time between the last two readings of the same tag was longer than 300 s, and can be made up of a single or several feeding events from different bunks, or when a new tag was detected in the same trough (Vytelle LLC, Calgary, AB, Canada). Meal events with a feed intake lower than 1 kg and time at bunk lower than 3 s were discarded. The following feeding behavior traits were analyzed: time spent at the feed bunk (TF, average daily time the animal spent at the feed bunk during the test period, min per day) and frequency of visits to the feed bunk (FF, average sum of feeding events of the animal per day, number of feeding events per day).

The phenotypic records were adjusted for the fixed effects listed in Table 3 using the *lm() function* in R. The adjusted phenotypes were then used for the association analyses.

**Table 3:** Effects included for phenotype adjustment for growth, feed efficiency, and feeding behavior traits

Trait^a^	Categorical fixed effects^b^	Covariates^c^
W210 (kg)	CGw	CA, CA^2^ AW
WSel (kg)	CGw	CA, CA^2^, ASW
ADG (kg.day^− 1^)	CGr, BM	AST
DMI (kg.day^− 1^)	CGr, BM	AST
RFI (kg.day^− 1^)	CGr, BM	AST
TF (hour.day^− 1^)	CGr, BM	AST
FF (n.day^− 1^)	CGr, BM	AST

^a^W210: weight at 210 days; WSel: body weight measured at the time of selection; ADG: average daily gain; DMI: dry matter intake; RFI: residual feed intake; TF: time spent at the feed bunk; FF: frequency of visits to the feed bunk

^b^CGw: contemporary group for weight (birth year, birth month, line, and sex); CGr: contemporary group for RFI (test start year, test start month, installation, and sex); BM: birth of month

^c^CA and CA^2^: cow age (linear and quadratic effects); AW: age at weaning; ASW: age at selection weight; AST: age at the start of the feeding test

### Genomic datasets

A total of 1,338 animals were genotyped with the GeneSeek Genomic Profiler HDi 75 K (GeneSeek Inc., Lincoln, NE, USA) panel containing 74,677 SNPs distributed along the genome, with a mean distance between markers equal to 32.3 ± 10 kilobases (Kb). The SNP positions were based on ARS-UCD1.2 genome [64]. The genotyped animals included 817 males, 519 females, and 2 founders (unknown sex). Non-autosomal SNPs, SNPs with unknow genome position, and SNPs with a GenCall score below 0.15 were removed during the genomic quality control. After the quality control, 69,680 SNPs remained for further analyses.

### Copy number variation identification

The CNV identification was performed using the PennCNV.1.0.5 software [65], which integrates Log R Ratio (LRR) and B Allele Frequency (BAF) on a per sample basis into a hidden Markov model to determine the number of copies and genotypes of each CNV. LRR measures the total signal intensity while BAF measures the proportion of the B allele in each sample. The population frequency of the B allele information was calculated using the BAF value of each SNP in all samples. To reduce false-positive results, the genomic waves were adjusted using the *-gcmodel* option in the PennCNV program. Genomic waves refer to a signal noise related to the GC content in the genome, which interferes with accurate CNV detection. The cattle *gcmodel* file was generated by calculating the guanine-cytosine (GC) content of each marker. The LRR values of each SNP were adjusted for the genomic waves along the genomic regions, taking into account the expected GC content in the bovine genome in a region of 500 Kb around each SNP and based on a regression model [66].

After CNV calling, a sample-based quality control was performed, which removed CNVs with a BAF drift lower than 0.01, standard deviation of LRR greater than 0.30, minimum length of 1,000 bp, maximum length of 5,000,000 bp, and GC wave factor lower than 0.05 (after genomic waves were corrected by guanine-cytosine content) to generate raw CNV calls. In addition, CNVs with less than three consecutive SNPs were discarded. Finally, after the control quality, 620 individuals and 3,161 CNV remained for further analyses.

### Copy number variation regions identification

The CNVR were determined by grouping the 3,161 CNVs that overlapped by at least 1 bp within each algorithm, using the *mergeBed* option of the BEDtools suite tool [67]. CNVRs were classified in deletions when the animal showed a region with loss of a chromosomal segment, duplication for repeated chromosomal regions, and mixed, when it was identified deletions and duplications in the same genomic region.

### Association analyses

The association analyses were performed considering only the CNVR identified in the autosomal chromosomes. The CNVRs were coded as -1 (deletion), 0 (neutral), and 1 (duplication). To test the potential associations between CNVRs with the pre-adjusted phenotypes, the model fitted is:

$$\text{y} = \text{Xb} + \text{Zu} + \text{e},$$

where **y** is the vector of pre-adjusted phenotypes for each trait; **b** is the fixed effect of the CNVR tested for potential association with the phenotype, **X** is a vector containing the genotype score for the tested CNVR; **u** is the random vector of polygenic effect with **u** ~ N(0, **A**σ_u_^2^), where **A** is the pedigree-based additive genetic relationship matrix, σ_u_^2^ is the additive genetic variance for each trait; **Z** is the incidence matrix for **u**; and **e** is a random vector of residual effects with **e** ~ N(0, **I**σ_e_^2^), where **I** is an identity matrix and σ_e_^2^ is the residual variance. The variance components were previously calculated by fitting the above-described model excluding the CNVR as a fixed effect in the model. The estimation of variance components and the CNVR effect size estimation were performed using the BLUPF90 + program [68].

The model adjusted provided the effect size (β) and standard error (SE) for each CNVR, which were used to compute the t statistic (t = β ⁄ SE). Subsequently, p-values were computed by assuming that a t-distribution with “n– 2” degrees of freedom, where n is the sample size (i.e., the number of animals used to obtain the CNVR effect for each pre-adjusted phenotype). To adjust for multiple testing, a Bonferroni correction at α = 0.05 genome-wise significance level was applied by dividing α by the total number of CNVRs.

### Gene annotation and functional analyses

The CNVRs significantly associated with the phenotypes were used for the annotation. The gene and QTL annotation in these regions was performed using the GALLO package [69], utilizing annotated data for *Bos taurus* sourced from the Ensembl database (www.ensembl.org/Bos_taurus/Info/Index) and the reference genome ARS-UCD1.2 [64]. Additionally, the Cattle QTL database (www.animalgenome.org/cgi-bin/QTLdb/BT/index) was used as a resource for obtaining previously-reported QTL information. To test the significance of the QTL representativeness, it was performed a QTL enrichment analysis using GALLO package. This analysis is based on a hypergeometric test approach, where the number of QTLs annotated within the candidate regions for each QTL type is compared with the observed number of QTLs in the reference database. Subsequently, The Database for Annotation, Visualization and Integrated Discovery (DAVID; version 6.8) [70] was used for conducting Gene Ontology (GO) and KEGG pathway enrichment (*p* < 0.05) analyses to identify biological processes, molecular functions, cellular components, and biological pathways associated with the positional candidate genes identified.

### Electronic supplementary material

Below is the link to the electronic supplementary material.

**Supplementary Material 1: Table S1.** Descriptive statistics of raw and adjusted phenotypes of growth, feed efficiency, and feeding behavior traits

**Supplementary Material 2: Table S2.** Copy number variation regions of deletion type associated with growth, feed efficiency, and feeding behavior

**Supplementary Material 3: Table S3.** Significant Gene Ontology (GO) terms and Kyoto Encyclopedia of Genes and Genomes (KEGG) pathway analyses

**Supplementary Material 4: Table S4.** Enriched quantitative trait loci (QTL) that overlapped with the genomic regions associated with DMI and FF

## Data Availability

The data supporting this study’s findings belongs to an experimental animal breeding program, and restrictions are applied to the availability of data. However, data are available by contacting the corresponding authors upon reasonable request and with permission of the program (contacting the researcher MEZM: mezmercadante@gmail.com).
