# Supplementary material for: Genome-wide association study between copy number variation and feeding behavior, feed efficiency, and growth traits in Nellore cattle
Source: BMC Genomics. 2024 Jan 11;25:54. doi: 10.1186/s12864-024-09976-8 (PMC10785391; doi:10.1186/s12864-024-09976-8)
Supplement: Supplementary file 1 — Supplementary Material 1: Table S1. Descriptive statistics of raw and adjusted phenotypes of growth, feed efficiency, and feeding behavior traits [file 12864_2024_9976_MOESM1_ESM.docx]

**Table S1.** Descriptive statistics of raw and adjusted phenotypes of growth, feed efficiency, and feeding behavior traits

|  | Nº of animals with phenotype | Phenotype | | |  | Adjusted Phenotype | | |
| --- | --- | --- | --- | --- | --- | --- | --- | --- |
| Trait^a^ |  | Minimum | Mean | Maximum |  | Minimum | Mean | Maximum |
| W210 (kg) | 1,314 | 90.1 | 199.5 | 320.5 |  | -232 | -0.04 | 72.3 |
| WSel (kg) | 1,306 | 137 | 331 | 512 |  | -163 | 2.47 | 139 |
| ADG (kg.d^-1^) | 734 | 0.286 | 1.054 | 1.69 |  | -0.689 | 0.001 | 0.618 |
| DMI (kg.d^-1^) | 734 | 3.68 | 8.48 | 12.6 |  | -3.53 | 0.002 | 3.54 |
| RFI (kg.d^-1^) | 734 | -2.19 | -0.007 | 3.17 |  | -2.21 | 0.002 | 2.99 |
| TF (hour.d^-1^) | 623 | 1.012 | 2.69 | 8.009 |  | -2.069 | 0.288 | 5.56 |
| FF (visit.d^-1^) | 623 | 8.081 | 24.6 | 91.8 |  | -21.8 | 6.018 | 60.95 |

^a^W210: weight at 210 days; WSel: body weight measured at the time of selection; ADG: average daily gain; DMI: dry matter intake; RFI: residual feed intake; TF: time spent on the feed bunk; FF: feed frequency.
