# Supplementary material for: Genome-wide association study between copy number variation and feeding behavior, feed efficiency, and growth traits in Nellore cattle
Source: BMC Genomics. 2024 Jan 11;25:54. doi: 10.1186/s12864-024-09976-8 (PMC10785391; doi:10.1186/s12864-024-09976-8)
Supplement: Supplementary file 2 — Supplementary Material 2: Table S2. Copy number variation regions of deletion type associated with growth, feed efficiency, and feeding behavior [file 12864_2024_9976_MOESM2_ESM.docx]

**Table S2.** Copy number variation regions of deletion type associated with growth, feed efficiency, and feeding behavior.

| **CNVR^a^** | **Chr^b^** | **Start (bp)** | **End (bp)** | **Type** | **Trait^c^** | **Effect** | **p_value** | **Ensembl ID** | **Gene ID** |
| --- | --- | --- | --- | --- | --- | --- | --- | --- | --- |
| CNVR1 | 2 | 135,110,420 | 135,653,313 | Mixed | FF | 0.9269 | 2.743x10^-6^ | *ENSBTAG00000000684*  *ENSBTAG00000048830*  *ENSBTAG00000008579*  *ENSBTAG00000045172*  *ENSBTAG00000038945*  *ENSBTAG00000012052*  *ENSBTAG00000012043*  *ENSBTAG00000002138*  *ENSBTAG00000043637*  *ENSBTAG00000003403*  *ENSBTAG00000008314*  *ENSBTAG00000008309*  *ENSBTAG00000003832*  *ENSBTAG00000003822* | *ARHGEF10L*  *RCC2*  *bta-mir-2358*  *PADI6*  *PADI4*  *PADI3*  *PADI1*  *5S_rRNA*  *PADI2*  *SDHB*  *ATP13A2*  *MFAP2*  *CROCC* |
| CNVR2 | 5 | 117,080,458 | 117,820,070 | Deletion | FF | 1.129 | 1.134x10^-9^ | *ENSBTAG00000012291*  *ENSBTAG00000044449* | *TBC1D22A*  *bta-mir-2285o-5* |
| CNVR3 | 6 | 116,755,758 | 117,164,372 | Duplication | FF | 9.878 | 3.232x10^-9^ | *ENSBTAG00000020108*  *ENSBTAG00000007164*  *ENSBTAG00000051271*  *ENSBTAG00000011044*  *ENSBTAG00000011042*  *ENSBTAG00000001328*  *ENSBTAG00000048974*  *ENSBTAG00000048789*  *ENSBTAG00000012577*  *ENSBTAG00000012575* | *LETM1*  *FGFR3*  *TACC3*  *TMEM129*  *SLBP*  *NKX1-1*  *UVSSA*  *MAEA* |
| CNVR4 | 7 | 10,092,268 | 10,174,209 | Duplication | FF | 3.406 | 1.092x10^-10^ | *ENSBTAG00000053498* |  |
| CNVR5 | 7 | 42,951,015 | 43,292,715 | Mixed | FF | 1.249 | 8.2x10^-7^ | *ENSBTAG00000000717*  *ENSBTAG00000019767*  *ENSBTAG00000048393*  *ENSBTAG00000047560*  *ENSBTAG00000016072*  *ENSBTAG00000039372*  *ENSBTAG00000050765*  *ENSBTAG00000039355*  *ENSBTAG00000010772*  *ENSBTAG00000002098*  *ENSBTAG00000002100*  *ENSBTAG00000016648*  *ENSBTAG00000015050*  *ENSBTAG00000015051*  *ENSBTAG00000027357*  *ENSBTAG00000014349*  *ENSBTAG00000003018*  *ENSBTAG00000021616* | *PLPP2*  *MIER2*  *THEG*  *C2CD4C*  *SHC2*  *ODF3L2*  *MADCAM1*  *TPGS1*  *CDC34*  *GZMM*  *BSG*  *HCN2*  *POLRMT*  *FGF22*  *RNF126*  *FSTL3*  *PRSS57* |
| CNVR6 | 7 | 43,359,066 | 43,823,809 | Mixed | FF | 3.727 | 1.821x10^-9^ | *ENSBTAG00000045828*  *ENSBTAG00000051609*  *ENSBTAG00000045829*  *ENSBTAG00000049418*  *ENSBTAG00000046105*  *ENSBTAG00000046188*  *ENSBTAG00000048122*  *ENSBTAG00000047217*  *ENSBTAG00000053281*  *ENSBTAG00000046406*  *ENSBTAG00000037418*  *ENSBTAG00000008607*  *ENSBTAG00000002434*  *ENSBTAG00000025597*  *ENSBTAG00000011351*  *ENSBTAG00000020764*  *ENSBTAG00000020766*  *ENSBTAG00000020772*  *ENSBTAG00000020776*  *ENSBTAG00000053003*  *ENSBTAG00000020780*  *ENSBTAG00000011639*  *ENSBTAG00000025233*  *ENSBTAG00000000550*  *ENSBTAG00000012172*  *ENSBTAG00000007480*  *ENSBTAG00000046542*  *ENSBTAG00000038221*  *ENSBTAG00000030839*  *ENSBTAG00000019419*  *ENSBTAG00000004112* | *PTBP1*  *PLPPR3*  *AZU1*  *U6*  *PRTN3*  *ELANE*  *CFD*  *MED16*  *U6*  *R3HDM4*  *KISS1R*  *ARID3A*  *WDR18*  *GRIN3B*  *TMEM259*  *CNN2*  *ABCA7*  *ARHGAP45*  *POLR2E*  *GPX4*  *SBNO2*  *STK11*  *CBARP*  *ATP5F1D*  *MIDN*  *CIRBP*  *FAM174C*  *EFNA2*  *PWWP3A*  *NDUFS7*  *GAMT* |
| CNVR7 | 8 | 15,562,312 | 15,781,720 | Duplication | FF | 4.541 | 2.007x10^-7^ |  |  |
| CNVR8 | 8 | 38,356,510 | 38,610,355 | Duplication | FF | 1.386 | 3.292x10^-6^ | *ENSBTAG00000020815*  *ENSBTAG00000011161*  *ENSBTAG00000011160*  *ENSBTAG00000018347* | *UHRF2*  *TPD52L3*  *IL33* |
| CNVR9 | 8 | 85,996,187 | 86,508,867 | Deletion | DMI | -0.156 | 4.10x10^-5^ | *ENSBTAG00000005092*  *ENSBTAG00000054391*  *ENSBTAG00000047649* | *ROR2*  *AUH* |
| CNVR10 | 9 | 2,637,837 | 2,700,411 | Mixed | FF | 5.616 | 9.307x10^-6^ |  |  |
| CNVR11 | 9 | 16,366,613 | 16,894,948 | Duplication | FF | 0.869 | 1.096x10^-10^ |  |  |
| CNVR12 | 9 | 15,312,685 | 15,469,154 | Duplication | FF | 6.097 | 4.583x10^-6^ | *ENSBTAG00000005869*  *ENSBTAG00000051278*  *ENSBTAG00000016751* | *SENP6*  *MYO6* |
| CNVR13 | 12 | 73,233,249 | 73,770,215 | Mixed | FF | 4.982 | 2.861x10^-9^ | *ENSBTAG00000004401*  *ENSBTAG00000039065* | *UGGT2*  *HS6ST3* |
| CNVR14 | 12 | 74,302,958 | 74,578,587 | Mixed | FF | 4.257 | 8.424x10^-7^ |  |  |
| CNVR15 | 12 | 64,618,237 | 64,736,496 | Duplication | FF | 6.909 | 2.611x10^-11^ |  |  |
| CNVR16 | 13 | 12,552,408 | 12,829,168 | Duplication | FF | 2.265 | 4.023x10^-11^ | ENSBTAG00000019488 | USP6NL |
| CNVR17 | 26 | 51,032,219 | 51,267,717 | Duplication | FF | 4.396 | 5.725x10^-6^ | *ENSBTAG00000009181*  *ENSBTAG00000054967*  *ENSBTAG00000051139*  *ENSBTAG00000017804*  *ENSBTAG00000050527* | *INPP5A*  *BNIP3* |

^a^Copy number variation region (CNVR) significantly (P < 0.005) associated with the traits

^b^Chromosome

^c^DMI: dry matter intake; FF: feed frequency
