# Supplementary material for: Genome-wide association study between copy number variation and feeding behavior, feed efficiency, and growth traits in Nellore cattle
Source: BMC Genomics. 2024 Jan 11;25:54. doi: 10.1186/s12864-024-09976-8 (PMC10785391; doi:10.1186/s12864-024-09976-8)
Supplement: Supplementary file 3 — Supplementary Material 3: Table S3. Significant Gene Ontology (GO) terms and Kyoto Encyclopedia of Genes and Genomes (KEGG) pathway analyses [file 12864_2024_9976_MOESM3_ESM.docx]

**Table S3.** Significant Gene Ontology (GO) terms and Kyoto Encyclopedia of Genes and Genomes (KEGG) pathway analyses.

| Category^a^ | Term | p-value^b^ | Trait^c^ |
| --- | --- | --- | --- |
| BP | GO:0036414 - Histone citrullination | 5.71x10^-9^ | FF |
| BP | GO:0043951 - Negative regulation of cAMP-mediated signaling | 0.00183 | FF |
| BP | GO:0009060 - Aerobic respiration | 0.00443 | FF |
| BP | GO:0036413 - Histone H3-R26 citrullination | 0.01178 | FF |
| BP | GO:0090630 - Activation of GTPase activity | 0.01670 | FF |
| BP | GO:0046038 - GMP catabolic process | 0.02919 | FF |
| BP | GO:0071260 - Cellular response to mechanical stimulus | 0.03143 | FF |
| BP | GO:0071321 - Cellular response to cGMP | 0.03492 | FF |
| BP | GO:0006909 - Phagocytosis | 0.03783 | FF |
| BP | GO:0006955 - Immune response | 0.00704 | FF |
| CC | GO:0005634 - Nucleus | 0.00278 | FF |
| CC | GO:0030133 - Transport vesicle | 0.02346 | FF |
| CC | GO:0005743 - Mitochondrial inner membrane | 0.04617 | FF |
| MF | GO:0004668 - Protein-arginine deiminase activity | 6.69x10^-5^ | FF |
| MF | GO:0004252 - Serine-type endopeptidase activity | 0.00691 | FF |
| MF | GO:0030246 - Carbohydrate binding | 0.01088 | FF |
| MF | GO:0003899 - DNA-directed 5'-3' RNA polymerase activity | 0.01185 | FF |
| PATHWAY | bta04740 - Olfactory transduction | 0.02403 | FF |
| PATHWAY | bta03420 - Nucleotide excision repair | 0.04908 | FF |

^a^BP: biological process; CC: cellular process; MF: molecular function; Pathway: metabolic pathaway

^b^Significance level: p < 0.05

^c^FF: feed frequency
